# Nurses' clinical leadership in the hospital setting: A systematic review

**DOI:** 10.1111/jonm.13570

**Published:** 2022-03-15

**Authors:** Carlota Guibert‐Lacasa, Mónica Vázquez‐Calatayud

**Affiliations:** ^1^ Clínica Universidad de Navarra Madrid Spain; ^2^ Clínica Universidad de Navarra Pamplona Spain; ^3^ Innovation for a Person‐Centred Care Research Group (ICCP‐UNAV) University of Navarra Pamplona Spain; ^4^ Navarra's Health Research Institute (IdiSNA) Pamplona Spain

**Keywords:** clinical leadership, clinical nurse, intervention, nursing, strategies

## Abstract

**Aim:**

This study aims to identify the most effective interventions to facilitate nurses' clinical leadership in the hospital setting.

**Background:**

There is a gap in the literature on the identification and measurement of effective interventions for leadership skill development among clinical nurses in hospitals. To the best of our knowledge, no systematic review has been performed on this issue.

**Evaluation:**

A systematic review was conducted. The PubMed, CINAHL, PsycINFO and Cochrane databases were reviewed. Data extraction, quality appraisal and narrative synthesis were conducted in line with Preferred Reporting Items for Systematic Reviews and Meta‐Analyses (PRISMA) guidelines.

**Key issues:**

The evidence reveals that interventions designed to promote nurses' clinical leadership are complex, requiring that cognitive, interpersonal and intrinsic competencies as well as psychological empowerment, emotional intelligence and critical reflexivity skills be addressed.

**Conclusions:**

The development of multicomponent, theory‐based and mixed‐format programmes may be more suitable to facilitate nurses' clinical leadership in the hospital setting.

**Implications for Nursing Management:**

Strategies to facilitate nurses' clinical leadership in the hospital setting should address simultaneously the knowledge and ability of bedsides nurses to solve the practical problem collaboratively with a sense of control, competency and autonomy. Hence, it would promote high quality care, satisfaction and retention of bedside nurses.

## 1 BACKGROUND

Clinical leadership is an ambiguous and context‐dependent concept (Larsson & Sahlsten, 2016). A growing body of literature has recently attempted to clarify this relatively new concept (Chávez & Yoder, 2015; Mianda & Voce, 2017; Stanley & Stanley, 2018). However, its meaning is still unclear, especially in the hospital context. For this review, nurses' clinical leadership refers to nurses who are directly involved in providing nursing care at the bedside and who exert influence on health care team colleagues to achieve positive patient outcomes, even though no formal authority has been vested in them (Chávez & Yoder, 2015; Patrick et al., 2011).

Nurse clinical leaders can be found across the spectrum of health organisations (Stanley & Stanley, 2018). In hospitals, where care is becoming more complex, with more demanding and high acuity patients, shorter lengths of stay and staffing shortages, nurses play a key leadership role (Daly et al., 2014). Nurses at the bedside are accountable for and oversee the completion of patient care as well as directly lead and manage the provision of safe patient care (Larsson & Sahlsten, 2016). They identify areas for improvement in advocating for patients and their families, motivate other members of the care team to act on patient care and lead change initiatives to solve problems that arise in daily clinical practice (Daly et al., 2014; Doherty, 2014). In addition, they identify inefficiencies in organisational structures, workflows, policies and procedures that affect the delivery of optimal patient care (Casey et al., 2011; Doherty, 2014; Patrick et al., 2011).

Promoting clinical leadership among frontline nurses is critical given their potential impact on patient outcomes and experiences (Aiken et al., 2011), team performance outcomes (O'Donovan et al., 2021), nurses' job satisfaction and retention (Chappell & Richards, 2015), quality, safety and effectiveness of care (Casey et al., 2011; Patrick et al., 2011).

According to the collaborative report between the Institute of Medicine (IOM) and the Robert Wood Johnson Foundation, the future of nursing depends on educating and supporting all levels of nurse leaders (IOM, 2011). For example, the International Council of Nursing (ICN), launched in 1995, the ICN LFC programme aims to prepare nurses with the leadership skills required to implement organisational change to improve nursing practice and achieve better health outcomes (Ferguson et al., 2016). Likewise, magnet hospitals, organisations that receive special designations for having created excellent nursing practice environments and providing excellent patient care, make significant investments in the clinical leadership development of their nursing staff (McCaughey et al., 2020). Despite these and other initiatives, several authors point out that nurses are not prepared to exercise leadership in hospital settings and call for effective strategies to prepare them for clinical leadership skills at the bedside (Curtis et al., 2011; Daly et al., 2014; Larsson & Sahlsten, 2016).

There is a gap in the literature on the identification and measurement of effective interventions for leadership skill development among clinical nurses in the hospital setting. To the best of our knowledge, no systematic review has been performed on this issue. Mianda and Voce (2018), in a recent systematic literature review, focused on interventions for clinical leadership among frontline health care providers without discriminating the context or participants, such as doctors and managers.

Nurses' leadership skills are acknowledged as playing an important role in the hospital setting and the health outcomes of patients (Daly et al., 2014). Thus, this systematic literature review will benefit the health sector and service consumers by identifying and evaluating evidence on effective interventions for the development of nurses' leadership skills. This knowledge will enable better utilization of resources and enhance programme development through the identification of the most effective interventions for leadership skill development for clinical nurses. Therefore, the objective of this systematic review was to identify the most effective interventions to facilitate nurses' clinical leadership in the hospital setting.

## 2 METHODS

### 2.1 Design

A systematic review of the most recent literature was carried out using the Preferred Reporting Items for Systematic Reviews and Meta‐Analyses (PRISMA) guidelines (Moher et al., 2009) for reporting.

### 2.2 Search methods

A systematic review of studies published in the PubMed, CINAHL, PsycINFO and Cochrane databases was performed in May 2021. For these electronic searches, as illustrated in Table 1, the terms ‘intervention’, ‘clinical leadership’, ‘nursing’ and their synonyms were combined with the Boolean operators ‘AND’ and ‘OR’. To improve search sensitivity and avoid omitting relevant studies, MeSH terms and the keywords identified in the selected studies were used. In particular, given the ambiguity of the term ‘clinical leadership’ and its recent use, different free terms used under the same umbrella and with the same meaning were included. The following limits were set: language, English and Spanish and year of publication within the last 10 years to ensure that the search was current.

**TABLE 1:** Search strategy

Search strategy				
Strategy OR Program* [Mesh Term] OR Intervention	AND	Staff Clinical Leadership OR Clinical Leadership OR Frontline Leadership OR Ward Leadership OR Clinician Leadership	AND	Nurs*[Mesh Term]

To complete the electronic searches, the ‘snowballing’ technique was applied by reviewing the reference lists of all selected studies and identifying possible additional papers. Manual reviews of the journals relevant to the area of interest were carried out: ‘Journal of Nursing Management’ and ‘Journal of Nursing Administration’.

Studies were selected based on the application of the inclusion and exclusion criteria presented in Table 2.

**TABLE 2:** Selection criteria for the studies

Inclusion	Exclusion
Experimental and quasi‐experimental studies on interventions that favour nurses' clinical leadership. Reviews on the subject with a rigorous systematic methodology, as long as the studies involved were not included in this review.	Descriptive or qualitative studies. Opinion articles. Studies that include among their participants students and/or other profiles (e.g., supervisors and advanced practice nurses). Studies carried out on nurses who work in outpatient or home settings.

### 2.3 Quality appraisal

The selected studies were independently evaluated by two authors (CGL and MVC) according to the methodological quality criteria described by PRISMA for systematic reviews, which includes 27 criteria (Urrútia & Bonfill, 2010), and by TREND for quasi‐experimental studies, which includes 22 criteria (Vallvé et al., 2005). The latter criteria were scored as ‘yes’, ‘no’, ‘unclear’, or ‘not applicable’. A total score was calculated by summing the ‘yes’ items, giving each study a score between zero and the total number of items evaluated in each checklist (i.e., 17, 18 or 19). Studies with a score equal to or lower than half of the items evaluated were considered to have a high level of bias and, therefore, poor methodological quality. Studies with a medium or high quality had higher scores. Disagreements between the two reviewers were resolved through discussion. No studies were excluded after evaluation. Due to the type of study identified, the risk of bias could not be assessed (Higgins et al., 2011).

### 2.4 Data abstraction

The characteristics of nurses' clinical leadership intervention programmes were categorized according to the competencies adapted from the American Organization for Nursing Leadership (AONL): ‘The Science’: cognitive, ‘The Art’: interpersonal and ‘The Leader Within’: intrinsic (AONL, 2015) and common components of nurse manager development programmes identified by Ullrich et al. (2021).

### 2.5 Synthesis

The data were analysed considering the research objectives, design and sample; the characteristics of the intervention; the instruments used to evaluate the intervention; and the main results of the studies reviewed. We synthesized the results through the formulation of interventions, the strategies used in interventions and the effectiveness of interventions in facilitating clinical leadership. This analysis process was first performed by the two researchers separately (CGL and MVC), and they then jointly compared, clarified and reached a consensus on the findings.

## 3 RESULTS

### 3.1 Search outcomes

**FIGURE 1:**
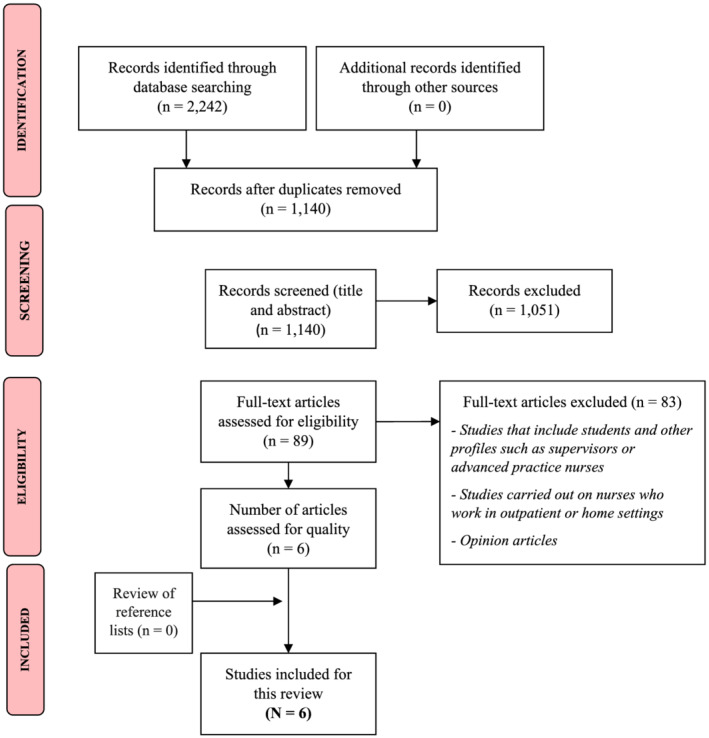
PRISMA flow diagram of the article selection process (Moher et al., 2009)

In the initial search, 2242 studies were identified. After removing duplicates (*n* = 102), the abstracts of 1140 articles were examined for their potential inclusion in the systematic literature review, of which 1051 were considered irrelevant for the purpose of this review. The remaining 89 articles were evaluated by two reviewers (CGL and MVC) independently and in full text, after which 83 articles were excluded for not meeting the inclusion criteria. Furthermore, no relevant article was found using the ‘snowball’ technique. Ultimately, six studies were included in this review (see Figure 1).

### 3.2 General study characteristics

Table 3 presents the main characteristics of the studies selected for this review. Of the six included articles, one was a systematic review, which included 17 pre–post studies, and the others were quasi‐experimental studies. Leggat et al. (2016) conducted their study in Australia, and the remaining five were carried out in the United States (Abraham, 2011; Chappell & Richards, 2015; Fitzpatrick et al., 2016; MacPhee et al., 2014; Shen et al., 2018).

**TABLE 3:** Characteristics of the studies selected in this review

Author, year and country	Design, sample and setting	Objective	Intervention				Instruments	Results	Quality
			Mechanism Programme, format, length and follow‐up	Competencies					
				A	B	C			
Chappell and Richards (2015) USA	Systematic review N: 22–1100 newly graduated nurses, predominantly female and <35 years N.R.	To evaluate the relationship between new graduate nurse and clinical leadership skills and new graduate nurse and clinical leadership skills transition programmes.	NGTP Mixed format: online and face to face Length: 12–24 months Follow‐up: N.R.	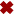		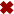	Leader Empowerment Behaviours Scale	Improved clinical leadership skills.^a^	PRISMA: 21/27
Leggat et al. (2016) Australia	Pre–post intervention study N: 62 health professionals (33 nurses) Long‐term care	To develop clinical leadership among health care professionals working in public sector organizations to improve their skills and ensure high quality and safe services.	EBL programme Mixed format: online and face to face Length: 12 months Follow‐up: N.R.	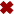	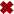	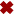	LPI Questionnaire Spreitzer Scale	Development of knowledge, skills and clinical leadership competencies.^a^ Improvement of psychological empowerment and emotional intelligence.^a^	Trend Statement: 18/22
MacPhee et al. (2014) USA	Quasi‐experimental study N1: 110; N2: 27 frontline nurses N.R.	To determine whether the NLI programme enhances leaders' use of empowerment behaviours.	NLI programme Format: online Length: 12 months Follow‐up: N.R.	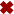	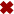	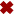	PES Questionnaire LEBS Questionnaire	Increased use of leader empowerment behaviours.^a^ Positive association between leader empowerment behaviours and feelings of structural empowerment.	Trend Statement: 19/22
Shen et al. (2018) USA	Quasi‐experimental study N: 36 nurses in four specialties and with 5–20 years of experience Acute and long‐term care; public health	To evaluate the effectiveness of the KNLR programme in improving nurses' knowledge and leadership skills.	KNLR programme Mixed format: online and face to face; individual and group Length: 6 months Follow‐up: 3 months	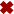	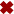	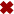	LKSI Questionnaire	Improvement of knowledge and leadership skills.^a^	Trend Statement: 18/22
Fitzpatrick et al. (2016) USA	Quasi‐experimental study N1: 33; N2: 35 bedside nurses with ≥1 year of experience N.R.	To determine the impact of the LEAD programme on the frequency of leadership practices and the importance of specific leadership behaviours perceived by participants.	LEAD programme format: face to face; individual and group Length: 6 months Follow‐up: 3 months	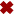	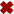	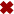	LPI Questionnaire	Improved frequency of leadership practices.^a^ Improvement in leadership importance ratings before and after the intervention.^a^	Trend Statement: 19/22
Abraham (2011) USA	Quasi‐experimental study N: 15 staff nurses with ≥5 years of experience N.R.	To evaluate the NLPP with respect to leadership skills and professional behaviours.	NLPP Format: face to face and group Length: 6 months Follow‐up: 6 months	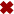		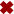	LPI Questionnaire NAS	Improvement of leadership skills.^a^ Changes in professional behaviour.^a^	Trend Statement: 17/22

Abbreviations: A, cognitive competencies; AHRQ, Agency for Healthcare Research and Quality level of evidence; B, interpersonal competencies; C, intrinsic competencies; CWEQII, Work Effectiveness Conditions Scale; EBL, Enquiry‐Based Learning; KNLR, Kansas Nurse Leader Residency; LEAD, Leadership Education and Development; LEBS, Leader Empowerment Behaviours Scale; LKSI, Knowledge Sharing Infrastructure; LKSI, Leadership Knowledge and Skills Inventory; LPI, Leadership Practices Inventory; N.R., not reported; NAS, Nursing Activity Scale; NGTP, New Graduate Nurse Transition Programme; NLI, Nursing Leadership Institute; NLPP, Nursing Leadership Perspectives Programme; PES, Psychological Empowerment Scale; USA, United States of America.

^a^ Statistically significant result.

### 3.3 Methodological quality of the studies

Table 3 details the methodological quality of the studies. In general, the studies presented medium quality (*n* = 4) and a lesser extent, high quality (*n* = 2). The most commonly found deficiencies, based on the criteria analysed for each type of study design, were related to the lack of theories used in designing behavioural interventions; insufficient description of the locations where data were collected; and lack of follow‐up, description and analysis of differences between groups in the follow‐up, among other factors.

### 3.4 Main findings of the studies

The analysis of the studies reviewed reveals various interventions aimed at promoting nurses' clinical leadership in the hospital context. For ease of understanding, the main findings are presented in three sections relating to the competencies addressed by the interventions, the mechanisms used and their evaluation.

### 3.5 Competencies of interventions to promote clinical leadership

Interventions to promote clinical leadership addressed three core competencies: (1) cognitive, (2) interpersonal and (3) intrinsic. Each of these is detailed below. The competencies of the interventions designed to promote nurses' clinical leadership are reflected in Table 3.

#### 3.5.1 Cognitive competencies

Cognitive competencies appear key to promoting nurses' clinical leadership, having been recurrently identified in programmes in five of the six studies reviewed (Abraham, 2011; Fitzpatrick et al., 2016; Leggat et al., 2016; MacPhee et al., 2014; Shen et al., 2018).

These competencies enable the development and application of knowledge for practical problem solving, making good decisions and controlling learning and behaviour (Abraham, 2011; Fitzpatrick et al., 2016; Leggat et al., 2016; MacPhee et al., 2014; Shen et al., 2018). More specifically, studies combine didactic and interactive learning through online and/or face‐to‐face training sessions (Leggat et al., 2016), discussion groups and role‐play activities to promote the translation of acquired leadership knowledge into practice (Chappell & Richards, 2015; Leggat et al., 2016; MacPhee et al., 2014; Shen et al., 2018).

Programmes that address these types of competencies improve nurses' decision‐making skills in daily practice, their ability to lead change within inpatient services (Fitzpatrick et al., 2016; Shen et al., 2018) and, ultimately, empower nurses at the bedside and equip them with the necessary skills for appropriate leadership with other health care professionals, patients and their families (Fitzpatrick et al., 2016). These competencies are therefore relevant in hospital settings because the bedside leaders identify practice improvement needs and bolster change.

#### 3.5.2 Interpersonal competencies

Other competencies identified as essential are interpersonal competencies, which refer to individual capacities and social skills with which to establish stable and effective relationships with other individuals, patients, families and professionals (Abraham, 2011; Fitzpatrick et al., 2016; Leggat et al., 2016; MacPhee et al., 2014; Shen et al., 2018). Strategies used to develop these competencies include mentoring and team reinforcement systems.

On the one hand, two of the studies reviewed include mentoring as a strategy to foster interpersonal relationships through individualized and informal sessions at the request of the person concerned (Leggat et al., 2016; MacPhee et al., 2014). The authors do not always specify what types of mentors were included in the interventions. However, generally, a mentor was defined as a person in a higher position and/or with more experience than the person involved. Programmes adopting this strategy, in addition to fostering relationships with other professionals, promoted the professional development of mentored persons and encouraged participants to take an active role in the organisation (Leggat et al., 2016; MacPhee et al., 2014).

On the other hand, four studies employed team reinforcement systems to strengthen workplace relationships (Fitzpatrick et al., 2016; Leggat et al., 2016; MacPhee et al., 2014; Shen et al., 2018). Most notably, Fitzpatrick et al. (2016) implemented a novel strategy called REJOICE (respect, empathy, individuality, collaboration and expression) to enhance the collegiality among inpatient service teams. This strategy included activities such as recognizing a co‐worker who had positively impacted someone's day, sharing experiences about nursing vocation and participating in committees and mentoring new staff (Fitzpatrick et al., 2016). These activities resulted in a shared vision and unified decision making, effective communication and an appropriate working environment that encouraged professional involvement and initiative within and across inpatient services (Leggat et al., 2016; Shen et al., 2018).

#### 3.5.3 Intrinsic competencies

These competencies are addressed in all of the studies reviewed (Abraham, 2011; Chappell & Richards, 2015; Fitzpatrick et al., 2016; Leggat et al., 2016; MacPhee et al., 2014; Shen et al., 2018), playing a crucial role in fostering the clinical leadership of care nurses.

Intrinsic competencies are closely related to the competencies described above; they reflect one's own values and determine the way in which a person positions him or herself and responds to the situations that arise. More specifically, the studies reviewed identify three intrinsic competencies that favour clinical leadership: (1) psychological empowerment (Leggat et al., 2016; MacPhee et al., 2014), (2) emotional intelligence (Leggat et al., 2016) and (3) critical reflexivity (MacPhee et al., 2014; Shen et al., 2018). It should be noted that although all programmes develop some of these competencies, there are no programmes in which all three appear.

Psychological empowerment refers to the ability of each nurse to have self‐control, make decisions and take responsibility for each of his or her actions and consequences. This skill was developed, for example, by means of the EBL programme through simulated classes and clinical cases (Leggat et al., 2016). Programmes developing this competency show a positive impact at the individual and organisational levels, leading to an improvement in nurses' self‐perception and commitment (Leggat et al., 2016; MacPhee et al., 2014) and to healthier working environments (MacPhee et al., 2014).

The second intrinsic skill, emotional intelligence, refers to nurses' ability to recognize, understand and exercise control over their own and others' emotions (Leggat et al., 2016). Leggat et al. (2016) adopted a programme of inquiry‐based learning to develop this competency through the use of clinical scenarios, where complex practice situations in which nurses had to take control and manage their emotions were simulated.

Finally, critical reflexivity refers to nurses' ability to be aware of themselves and their influence on others (MacPhee et al., 2014; Shen et al., 2018). To develop this competency, one‐to‐one mentoring was used in two studies (MacPhee et al., 2014; Shen et al., 2018) for the acquisition of skills, knowledge and subsequent reflexivity in daily practice for a relationship of trust established between the participant and mentor.

### 3.6 Mechanisms to promote clinical leadership

The main mechanisms shaping interventions to promote clinical leadership are described below in terms of programme development frameworks, delivery formats, programme durations and types, recipients and organisational support.

### 3.7 Programme development framework

Of the articles reviewed, half specify the theories underpinning the design of nurse clinical leadership interventions (Abraham, 2011; MacPhee et al., 2014; Shen et al., 2018), and the other half are not explicit (Chappell & Richards, 2015; Fitzpatrick et al., 2016; Leggat et al., 2016). Shen et al. (2018) allude to the learning domain framework for the development of the nursing clinical leadership programme, which is based on the AONL competency model. MacPhee et al. (2014) draw on the conceptual framework of psychological empowerment of leadership based on social psychological theory to develop their programme. Similarly, Abraham (2011) frames his intervention within Ernest Boyer's theory, which serves as a guide for linking programme objectives to the learning activities experienced by each professional.

#### 3.7.1 Administration format, duration and type of programme

Interventions to promote nurses' clinical leadership use different modalities in terms of the delivery formats, durations and types of programmes used, as shown in Table 3. Two of the selected studies involved face‐to‐face interventions (Abraham, 2011; Fitzpatrick et al., 2016). Face‐to‐face programmes are those in which the provider is in direct physical contact with the recipient. Among these programmes are the Leadership Education and Development (LEAD) programme and Nursing Leadership Perspectives Programme (NLPP), which consist of six 4‐h individual and group sessions held over a period of 3–6 months. The LEAD programme focuses on developing skills to empower nurses as clinical leaders (Fitzpatrick et al., 2016), while the NLPP is based on an educational model developed to enhance leadership skills and promote the professionalism of registered nurses at the bedside (Abraham, 2011). The programme aims to enhance leadership skills, promote professional nursing activities and improve the understanding of professional nursing, shared decision making and interdisciplinary collaboration (Abraham, 2011).

One of the studies covered an online intervention programme that did not involve in‐person attendance (MacPhee et al., 2014). Specifically, workshops involving four training sessions on didactic and interactive learning were held over 1 year. The leadership development programme based on an NLI empowerment framework developed by MacPhee et al. (2014) uses this modality. This programme enables the improvement of leadership behaviours and performance. From a training perspective, the programme focuses specifically on training and applications for leader empowerment behaviours. These behaviours can be taught, assessed or measured (MacPhee et al., 2014).

Finally, three studies (Chappell & Richards, 2015;Leggat et al., 2016; Shen et al., 2018) included interventions delivered in a blended form focused on the Kansas Nurse Leader Residency (KNLR), the New Graduate Nurse Transition Programme (NGTP) and Enquiry‐Based Learning (EBL) combining face‐to‐face and online formats. The duration of these interventions ranged from 6 to 24 months: NGTP ranged from 12 to 24 months, and NLI and EBL lasted 12 months. The EBL programme comprised a workplace project, whereby participants were required to identify, plan, implement and evaluate a quality or safety initiative in their workplace. For example, some of these initiatives included multidisciplinary handovers to reduce clinical incidents or improvements in clinical assessment recording to improve patient flow within the hospital (Leggat et al., 2016). The KNLR programme addressed the development of nurses' knowledge and leadership skills in science, art and creating leaders while also working on quality and safety through small change projects. Clinical nurses carried out small change projects to reduce, for example, failed intravenous injection attempts, the rate of catheter‐associated urinary tract infections and the incidence of falls among older adult patients (Shen et al., 2018), all of which are relevant to inpatient services.

#### 3.7.2 The recipients of the intervention and context of application

Most of the studies included focused interventions on care nurses (Abraham, 2011; Chappell & Richards, 2015; Fitzpatrick et al., 2016; MacPhee et al., 2014; Shen et al., 2018), while only one study included other health professionals (Leggat et al., 2016).

The profiles of the care nurses surveyed also varied in terms of years of professional experience, ranging from recent graduates (Chappell & Richards, 2015) to those with 1 year (Fitzpatrick et al., 2016) and those with at least 5 years of professional experience (Abraham, 2011). Shen et al. (2018) and Leggat et al. (2016), while not specifying the years of experience of the studied nurses, noted that they had to have a high degree of expertise in the particular area of work.

Although all interventions have been carried out in hospital settings, two specify that they have been carried out in the acute and/or long‐term context without describing the study setting in more detail (Leggat et al., 2016; Shen et al., 2018).

#### 3.7.3 Organisational support

Four of the included studies emphasize the importance of organisational support for the implementation of programmes that promote the clinical leadership of bedside nurses without providing much detail about the type of this support provided (Abraham, 2011; Leggat et al., 2016; MacPhee et al., 2014; Shen et al., 2018). Shen et al. (2018) mention that they gave the grant to support the funding of the leadership programme. Leggat et al. (2016) offer a variety of media, such as online videos and webinars, to assist participants in their learning sets. Briefly, MacPhee et al. (2014) declare that institutions must provide opportunities in the workplace for the consequent construction of leaders to flourish within their practice environments. Therefore, one of the proposed initiatives is to release time for project work and online knowledge networks to facilitate connections among professionals. Another type of support alluded to by Abraham (2011) would be structural support by the creation of committees, workgroups or councils to facilitate the active participation of these nurses.

### 3.8 Effectiveness of clinical leadership interventions

To assess the effectiveness of these interventions at promoting the clinical leadership of nurses, it is necessary to have valid and reliable measurement instruments. For the purposes of this paper, any type of questionnaire, scale, test or functional test used to assess the interventions described above is considered an instrument.

Numerous instruments have been identified in the literature reviewed, whose contexts of application, reliability and dimensions identified for their operationalization are described in Table 4. Most of them were generic, either because of the context in which they are developed, inpatient or outpatient settings, or because of the discipline of application. The instruments used were validated and demonstrated excellent reliability, as shown in Table 4. However, none assessed all the competencies and skills that have been considered key in a clinical leadership intervention. It should be noted that in addition to these instruments, no studies included patient outcome measures that reflect whether the interventions to foster nurses' clinical leadership were effective at the level of safety and quality.

**TABLE 4:** Instruments used in the reviewed studies

Instruments	Competencies			Context	Operationalization	Reliability
	A	B	C			Cronbach's alpha
LPI (Abraham, 2011; Fitzpatrick et al., 2016; Leggat et al., 2016)	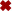	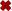	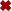	Generic	Measures leadership ability and leadership behaviours and contains five subscales: Challenging the Process, Inspiring a Shared Vision, Shaping the Way, Stimulating the Heart and Allowing Others to Act.	.95
Spreitzer Scale (Leggat et al., 2016)			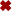	Generic	Measures psychological strengthening or empowerment.	.86
CWEQII (MacPhee et al., 2014)			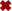	Generic	Measure structural empowerment.	.88
PES (MacPhee et al., 2014)			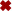	Generic	Measures psychological empowerment: meaning, competence, self‐determination and impact.	.81
LEBS (MacPhee et al., 2014)			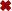	Generic	It measures the empowering behaviours of leaders: meaningful work, participation in decision making, employee confidence, facilitating the achievement of goals and autonomy from the bureaucracy.	.95
LKSI (Shen et al., 2018)	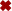	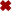	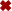	Nursing	It measures three main areas of leadership knowledge and skills.	—
NAS (Abraham, 2011)	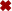			Nursing	Measures professional nursing activities.	.99

Abbreviations: A, cognitive; B, interpersonal; C, intrinsic; CWEQII, Work Effectiveness Conditions Scale; LEBS, Leader Empowerment Behaviours Scale; LKSI, Leadership Knowledge and Skills Inventory; LPI, Leadership Practices Inventory; NAS, Nursing Activity Scale; PES, Psychological Empowerment Scale.

In this regard, and given the heterogeneity of competencies, mechanisms and instruments used to assess the results, it is not possible to determine which intervention is more effective in facilitating clinical leadership. Despite this, it should be mentioned that in all the studies, significant improvements were obtained after implementing the intervention in terms of knowledge, skills and leader empowerment behaviours (Abraham, 2011; Chappell & Richards, 2015; Fitzpatrick et al., 2016; Leggat et al., 2016; MacPhee et al., 2014; Shen et al., 2018). With regard to the knowledge and skills, improvements were obtained in decision making, negotiation and communication skills (Shen et al., 2018). Concerning the empowerment behaviours, the study conducted by Leggat et al. (2016) obtained significant improvement after the programmes completion in emotional intelligence (*t* = 2.923; *df* = 109.7; *p* = .004) and the one by MacPhee et al. (2014) in leader empowering behaviour (*t* = 7.75; *df* = 0.06; *p* < .001) and psychological empowerment (*t* = 3.31; *df* = 0.12; *p* < .001). Similarly, Abraham (2011) obtained a significant change in professional behaviour after participation in the programme. For instance, they described an increased leadership involvement of the participants in their unit and departmental committees, workgroups and councils. Other empowerment behaviours were publishing an article, beginning a research study and leading practice initiatives as staff nurses to improve the quality and safety of patient care (Abraham, 2011).

## 4 DISCUSSION

This review identified what competencies and mechanisms should be addressed in interventions to facilitate nurses' clinical leadership in the hospital setting. In addition, some instruments to measure their effectiveness are suggested.

The need to include cognitive, interpersonal and intrinsic competencies in these interventions is a finding consistent with a previous integrative review in which a nurse's clinical leader is described as demonstrating three attributes: clinical competence and expertise, skills for building teams and relationships and personal qualities (Mannix et al., 2013). This result, however, could not be compared with those obtained in a recent systematic review (Mianda & Voce, 2018) focused on interventions for clinical leadership among frontline health care providers because the specific competencies to be developed are not identified. Moreover, as previously mentioned, Mianda and Voce (2018) describe the results without discriminating against the context, inpatient from outpatient settings, or participants such as doctors and managers. Given that clinical leadership remains an ambiguous and unclear concept (Chávez & Yoder, 2015; Mianda & Voce, 2017; Stanley & Stanley, 2018), knowledge of core competencies will provide a common understanding to guide the further development of effective interventions and tools to measure the clinical leadership of nurses in the hospital setting (Larsson & Sahlsten, 2016). Nevertheless, as the leadership role is influenced by context (Larsson & Sahlsten, 2016), further research is essential to identify characteristic distinctions in disparate settings.

In this sense, an interesting result of this review is that although the three competencies outlined have been identified in most of the programmes examined, they are only partially addressed. For instance, concerning intrinsic competencies, none of the studies reviewed included all of these skills identified (Abraham, 2011; Chappell & Richards, 2015; Fitzpatrick et al., 2016; Leggat et al., 2016; MacPhee et al., 2014; Shen et al., 2018). The findings of this review add knowledge to previous work on the three skills a clinical nurse leader should be prepared for psychological empowerment, emotional intelligence and critical reflexivity. It is worth mentioning that, due to the shortage of literature and the methodological limitations of these studies, there may be competencies that have not been identified and others that need to be explored in greater depth. For example, the knowledge and ability of bedside nurses to solve a practical problem collaboratively could be further explored (McCaughey et al., 2020). These results can be attributed to the lack of consensus in the definition of nurses' clinical leadership (Chávez & Yoder, 2015; Mianda & Voce, 2017; Stanley & Stanley, 2018) and the need for further research on this issue guided by a coherent theory‐based framework (MacPhee et al., 2012).

On the basis of the results of this review, in which only half of the studies selected specified the programme development framework (Abraham, 2011; MacPhee et al., 2014; Shen et al., 2018), the AONL competency model is proposed as a valuable framework for outlining the essential knowledge, skills and ability that successful nurse clinical leaders should possess. This competency model has been used extensively in nursing leadership development (AONL, 2015; Sherman & Pross, 2010) but less for nurses' clinical leadership (Shen et al., 2018) and could provide a guide, together with the results of this review, for further interventions in the hospital setting. According to MacPhee et al. (2012), a nursing leadership development programme with a strong theory‐based framework will lead to sustainable positive outcomes.

The results of the present review broaden this notion because they not only identify the competencies necessary to develop but also suggest different strategies to be used to develop each of the three core competencies: didactic and interactive learning strategies to develop cognitive competencies, mentoring and team reinforcement to acquire interpersonal competencies and experiential learning to develop intrinsic competencies (Abraham, 2011; Chappell & Richards, 2015; Fitzpatrick et al., 2016; Leggat et al., 2016; MacPhee et al., 2014; Shen et al., 2018). In this sense, integrating methodologies such as simulations, role playing and case studies (Vázquez‐Calatayud et al., 2017) into training may be interesting, which may allow nurse to improve their clinical leadership competencies and, for instance, to empower them to participate in the design and development of improvements that emerge bottom‐up.

Most studies use mixed interventions (Chappell & Richards, 2015;Leggat et al., 2016; Shen et al., 2018) that combine face‐to‐face and online formats as well as individual and group sessions to increase levels of effectiveness over time. It is important to consider this finding when choosing the most appropriate format for intervening with clinical nurses because using a single format could make it difficult to involve a workforce of professionals from different generations with different values and needs (Vázquez‐Calatayud, Errasti‐Ibarrondo, & Choperena, 2021). In addition, in the hospital setting, with limited time and the difficulty of being absent during the work shift as barriers to participating in professional development activities, e‐learning may address these limitations and those commonly associated with the COVID‐19 situation in future programmes. Therefore, it is appropriate not only to adequately screen staff but also to ask staff their motivations and adapt to the requests of clinical nurses for their subsequent participation in a programme. The profiles of the nurses involved also varied in terms of years of professional experience (Abraham, 2011; Chappell & Richards, 2015; Fitzpatrick et al., 2016). Some controversy has been found in terms of the experience advised for preparation as leaders. Some authors advise preparing nurses with some degree of experience and expertise in the given service (Leggat et al., 2016; Shen et al., 2018). This finding coincides with the assumptions that defend the theory developed by Patricia Benner (1982) on nursing expertise. At first, nurses need opportunities to develop and refine their clinical skills. Once they have become competent in practice, they are open to developing new and more complex competencies, such as intrinsic competencies. Others point to the need to focus the preparation of nurses on leadership competencies much earlier (Chappell & Richards, 2015). In this regard, it is necessary to point out that for nurses to develop their full potential as clinical leaders, their preparation is fundamental. Rarely are nurses trained in the intrinsic competencies that are key to dealing with the many situations they face in daily practice. A clear example is the global pandemic that nursing students and professionals have had to cope with (Mohebbi & Eslami, 2021; Vázquez‐Calatayud, Rumeu‐Casares, et al., 2021).

It is worth mentioning that in all studies reviewed, significant improvements were achieved after implementing the intervention in terms of knowledge, skills and behaviours in leadership and/or psychological and emotional empowerment (Abraham, 2011; Chappell & Richards, 2015; Fitzpatrick et al., 2016; Leggat et al., 2016; MacPhee et al., 2014; Shen et al., 2018). The improvement in knowledge and leadership skills reported by all studies, and in line with Larsson and Sahlsten (2016), is considered key to gaining the trust of others and positively impacting quality and patient safety. As bedside nurses occupy an informal leadership position, the trust placed in them is essential. Nurses gain confidence in bedside nurses as leaders when they demonstrate their knowledge of practical problem solving, good decision making, learning and behaviour management (Fitzpatrick et al., 2016; Leggat et al., 2016; MacPhee et al., 2014; Shen et al., 2018), enabling them to achieve a certain status or authority as leaders (Larsson & Sahlsten, 2016).

The improvement of psychological and emotional empowerment is considered another outcome of great interest. According to the recent study by Khoshmehr et al. (2020), better psychological empowerment may lead to reduced mental pressures and work environment stressors and enhance decision‐making power and moral behaviour performance by nursing staff, ultimately resulting in the creation of moral courage in nurses. In these circumstances, nurses can properly manage complex situations in daily practice, which is more common in the hospital setting. By having a sense of control, competency and autonomy, they feel more motivated and satisfied, which has a positive impact on retention and the quality of care provided (Khoshmehr et al., 2020; Marufu et al., 2021). This has been particularly crucial during the coronavirus outbreak to improve job performance (Mohebbi & Eslami, 2021). Concerning quality and patient safety, it is striking that, as Leggat et al. (2016) acknowledge, no reviewed study has included clinical outcome measures that reflect whether the programmes had a real impact on both.

Finally, it should be mentioned that none of the studies reviewed has used an instrument that comprehensively measures clinical leadership. This result can be attributed to the lack of consensus in the definition of nurses' clinical leadership (Chávez & Yoder, 2015; Mianda & Voce, 2017; Stanley & Stanley, 2018). However, it should be noted that among the available questionnaires, there is one used in different contexts, with high validity and reliability, to measure leadership ability and leadership behaviours, the ‘Leadership Practices Inventory’ (LPI) (Kouzes & Posner, 2017), which is close to its definition. This questionnaire includes five subscales that partially cover the three competencies identified in this review and, together with the Psychological Empowerment Scale (PES), could be used to evaluate interventions that favour nurses' clinical leadership.

### 4.1 Limitations

Some limitations of this review should be considered. Studies may have been omitted from the review if they were not published in the databases analysed or if they were published in languages other than English or Spanish. This review was further limited by weaknesses in study design because the studies were quasi‐experimental pretest and post‐test design, which is considered Level III evidence (Vallvé et al., 2005), implying that the randomized controlled studies were limited. Therefore, future studies should adopt designs that provide more rigorous evidence. Moreover, the use of self‐administered questionnaires for data collection in all studies may have led to social desirability bias, with participants providing scores that they felt were more acceptable to the researchers. Future studies could also use the triangulation method to thoroughly examine the effectiveness of leadership programmes in order to understand more clearly the dynamics of the leading process through qualitative and quantitative evaluations. The small sample size of the majority of studies is another limitation in interpreting the results. Difficulty in recruiting participants is pointed out in several studies selected due to limited funds and resources to recruit large numbers and sustain programmes. In addition, the studies are conducted in a single setting. The inclusion of other hospitals would probably have enriched both the number of participants and the heterogeneity of the sample, as the context could have influenced the bedside nurses' clinical leadership. Most studies were conducted in the United States, reflecting a gap in research and potentially omitting programmes offered in other countries, which limited the study results and inferences. This study also has several strengths, including its rigorous search for and selection of articles, thorough analysis of the literature, detailed description of results and important implications for practice.

## 5 CONCLUSIONS

This systematic review of the literature provides relevant information to support the design, implementation and evaluation of further leadership programmes. Based on the results of this review, it is suggested that the development of multicomponent, theory‐based, AONL frameworks and mixed‐format programmes may be more suitable to facilitate nurses' clinical leadership in the hospital setting. Multicomponent programmes should address cognitive, interpersonal and intrinsic competencies, as well as psychological empowerment skills, emotional intelligence and critical reflexivity. There is a clear need for further development of nurses' clinical leadership instruments to comprehensively evaluate these programmes. In the meantime, the combination of two valid and real tools, such as LPI and PES, could be useful.

## 6 IMPLICATIONS FOR NURSING MANAGEMENT

The knowledge provided by this review will help enlighten nurse managers and lectures to design educational and management strategies directed at developing competent clinical nurse leaders in the hospital setting and subsequently at enhancing the quality of care, satisfaction and retention of bedside nurses. In particular, theory‐based, mixed‐format and multicomponent programmes should address simultaneously the knowledge and ability of bedsides nurses to solve the practical problem collaboratively with a sense of control, competency and autonomy. Hence, these programmes may, for example, help nurses to actively participate in committees and working groups, propose projects to improve daily practice in the units or have a voice in multidisciplinary team rounds.

This review will also serve as a starting point to define the focus of future interventions. Determining the effectiveness of nurses' clinical leadership for patients through intervention‐type studies that include clinical outcome indicators could demonstrate the importance of investing in clinical leaders at the bedside in health care organisations.

## CONFLICT OF INTEREST

No conflicts of interest have been declared by the authors.

## AUTHOR CONTRIBUTIONS

CGL and MVC made substantial contributions to conception and design, acquisition of data or analysis and interpretation of data; are involved in drafting the manuscript or revising it critically for important intellectual content; and given final approval of the version to be published. Each author should have participated sufficiently in the work to take public responsibility for appropriate portions of the content. CGL and MVC agreed to be accountable for all aspects of the work in ensuring that questions related to the accuracy or integrity of any of the work are appropriately investigated and resolved.

## Supporting information




**Data S1.** Supporting Information

## Data Availability

Authors do not wish to share the data.
